# Participation in gardening activity and its association with improved mental health among family caregivers of people with dementia in rural Uganda

**DOI:** 10.1016/j.pmedr.2021.101412

**Published:** 2021-05-30

**Authors:** Herbert E. Ainamani, Wilson M. Bamwerinde, Godfrey Z. Rukundo, Sam Tumwesigire, Rebecca M. Kalibwani, Evard M. Bikaitwaho, Alexander C. Tsai

**Affiliations:** a Department of Mental Health, Kabale University School of Medicine, Kabale, Uganda; b Department of Environment and Natural Resources, Kabale University, Kabale, Uganda; c Mbarara University of Science and Technology, Mbarara, Uganda; d Department of Pediatrics, Kabale University School of Medicine, Kabale, Uganda; e Department of Agriculture, Bishop Stuart University, Mbarara, Uganda; f Department of Public Health Kabale University School of Medicine, Kabale, Uganda; g Center for Global Health and Mongan Institute, Massachusetts General Hospital, Boston, United States

**Keywords:** ADRD, Alzheimer’s disease and other related dementias, BADLS, Bristol Activities of Daily Living Scale, LAMICs, Low- and middle-income countries, ZBI, Zarit Burden Interview, anxiety, caregiving burden, dementia, depression, gardening, mental health, sub-Saharan Africa, Uganda

## Abstract

Evidence from high-income settings suggests that gardening is associated with reductions in depression, anxiety, and stress. The benefits of gardening are less well understood by mental health practitioners and researchers from low- and middle-income countries. Our study estimated the association between participation in gardening and symptoms of depression, anxiety, and stress among caregivers of people living with dementia in rural, southwestern Uganda. In a cross-sectional study, we interviewed 242 family caregivers of people with dementia to elicit their gardening activities; symptoms of depression, anxiety, and stress (Depression Anxiety Stress Scales); and caregiving burden (Zarit Burden Interview). Linear multivariable regression models estimated the association between participation in gardening and symptoms of depression, anxiety, and stress. Out of 242 participants, 131 (54%) caregivers were involved in gardening. Severe to extremely severe symptoms of depression were less prevalent among those who were involved in gardening compared with those who were not (0 [0%] vs. 105 [95%], P < 0.001), as were severe to extremely severe symptoms of anxiety (36 [27%] vs. 110 [99%], P < 0.001) and stress (2 [2%] vs. 94 [85%], P < 0.001). In regression models adjusting for covariates,we found statistically significant associations between participation in gardening and symptoms of depression (b = -18.4; 95% CI, 20.5 to −16.3), anxiety (b = -16.6; 95% CI, −18.6 to –14.6), and stress (b = -18.6; 95% CI, −20.6 to –16.6). Caregivers of people with dementia who participate in gardening have lower symptoms of depression, anxiety, and stress. Gardening interventions in this at-risk population may ameliorate symptoms of depression, anxiety, and stress.

## Highlights

• Family caregivers of people living with dementia experience high levels of anxiety and stress.
• Participating in gardening is associated with lower symptoms of depression, anxiety and stress.
• These findings may provide insights into future gardening intervention trials.

## 1 Introduction

With the increasing numbers of people with Alzheimer’s disease and other related dementias (ADRD) and concomitant increases in caregiving burden for family caregivers, the provision of mental health services for family caregivers will be among the most significant challenges for mental health systems in low and middle-income countries (LAMICs). In these settings, mental health systems are poorly funded, and caregiving responsibilities for people with dementia are borne primarily by family members (Ainamani et al., 2020, Ferrara et al., 2008, Werner et al., 2012). These challenges are further compounded by food and water insecurity (de Jager et al., 2017, George-Carey et al., 2012, Gurayah, 2015, Johnston et al., 2020, Mkhonto and Hanssen, 2018, Olakehinde et al., 2019). Exacerbating the burden of mental ill-health among family caregivers of people with ADRD are low levels of awareness about dementia and lack of access to high quality health care services (Kigozi et al., 2010, Owokuhaisa et al., 2020, Wakida et al., 2018).

Studies from high-income countries have suggested mental health benefits accruing from involvement in various forms of gardening (Barton and Rogerson, 2017, Clatworthy et al., 2013, Gonzalez and T., Patil, G., Martinsen, E., & Kirkevold, M. , 2011, Gonzalez et al., 2009, Kam and Siu, 2010, Stepney and Davis, 2005). A meta-analysis of the health effects of gardening and horticulture revealed a wide range of health benefits, including reductions in depression, anxiety, and body mass index, and increases in life satisfaction, quality of life, and self-esteem (Soga et al., 2017). Consistent with this literature, a systematic review that assessed the effectiveness of farm-based interventions for people with psychiatric problems recommended that farm-based interventions should be included in standard mental health treatment packages (Iancu et al., 2015).

The relationship between food insecurity and mental health problems is well known (Cooper-Vince et al., 2018, Dewing et al., 2013, Perkins et al., 2018, Tsai et al., 2012, Tsai et al., 2016b). However, the potential benefits of gardening activity are less well understood by mental health practitioners, researchers and policy makers from low- and middle-income countries. Our study sought to estimate the association between participating in gardening and symptoms of depression, anxiety and stress among caregivers of people living with dementia in a rural region of southwestern Uganda. We hypothesized that family caregivers who actively participate in gardening would have fewer symptoms of depression, anxiety and stress compared with caregivers who do not participate in gardening.

## 2 Methods

### 2.1 Population and design

In this cross-sectional study, we used surveys to assess caregivers’ involvement in gardening and symptoms of depression and anxiety. Our sample consisted of 242 family caregivers of patients with ADRD who were engaged in care at two health centers in the districts of Rukiga and Rubanda in southwestern Uganda. The local economy is primarily characterized by subsistence agriculture, animal husbandry, and small scale trading, with significant pockets of food and water insecurity (Tsai et al., 2012, Tsai et al., 2016a).

### 2.2 Recruitment and sampling procedure

Using convenience sampling, we collected data on 242 family caregivers between July and August 2020. We enrolled adult family caregivers of older-age people living with dementia who lived in the same house or compound with their patients and who had been involved in caregiving for more than 6 months. (In the remainder of this manuscript, we use the term “patients” to refer to people living with dementia, acknowledging that their caregivers were not formally involved with their care in a health worker capacity). Health workers from two specialized medical centers; Reach One Touch One Ministries (Rukiga) and Heal Medical Centre (Rubanda) helped our research team identify older-age people with dementia and their caregivers. We excluded caregivers who were unable to provide information about their patients due to physical or cognitive challenges such as deafness/mutism or acute intoxication.

Survey instruments were translated from English to Runyankore-Rukiga, the local language. Both oral and written informed consent were obtained from each study participant. Participants who did not know how to read and write were asked to provide a fingerprint to indicate consent. Consistent with local etiquette, we provided each participant with 2 bars of soap and a 1 kg package of salt for their participation.

### 2.3 Ethical considerations

Ethical approval for this study was obtained from the Mbarara University of Science and Technology Research Ethics Committee (SS4893). Further clearance to conduct this study was obtained from the Uganda National Council for Science and Technology and the Research Secretariat in the President’s office.

### 2.4 Survey assessment

Following prior work (Litt et al., 2017), we elicited gardening activity by asking caregivers whether they physically participate in gardening using their hands. The Depression, Anxiety and Stress Scales (DASS) were used to assess caregivers’ mental health problems (Lovibond and Lovibond, 1995). The depression, anxiety, and stress subscales each contain 14 items, each of which is scored on a 4-point Likert-type scale (“never”, “sometimes”, “often”, “almost always”), yielding a total score ranging from 0 to 42. Higher DASS scores indicate more severe symptoms of depression, anxiety, and/or stress. The DASS has been used in comparable samples and has shown adequate internal consistency (Ainamani et al., 2020, Crawford and Henry, 2003, Gloster et al., 2008). In our sample, the Cronbach’s α was 0.89. Prior work has derived the following thresholds for severity of depressive symptoms: normal (≤9), mild (10–13), moderate (14–20), severe (21–27), and extremely severe (≥28). The relevant thresholds for severity of anxiety symptoms are as follows: normal (≤7), mild (8–9), moderate (10–14), severe (15–19), and extremely severe (≥20).

We measured caregiving burden using the Zarit Burden Interview (ZBI) (Zarit et al., 1980). The 22-item scale measures different aspects of caregiving burden, each of which is scored on a 5-point Likert scale that ranges from “never” to “nearly always,” yielding a total sum score that ranges between 0 and 88. The ZBI is frequently used in research studies to assess caregiving burden and its association with mental health problems (Ainamani et al., 2020, Springate and Tremont, 2014). Patient functional status was assessed using the Bristol Activities of Daily Living Scale (BADLS) (Bucks et al., 1996). BADLS total scores range from 0 to 60, with higher scores indicating more severe functional impairments (Ainamani et al., 2020, Jefferson et al., 2008). Additional covariates included age and sex of the caregivers and their patients, the caregivers’ relationships with their patients, caregivers’ educational attainment (in years), and total duration of caregiving (in years).

### 2.5 Data analysis

We compared the characteristics of people who engaged in gardening activities vs. those who did not, using chi-square-tests for categorical variables and t-tests for continuous variables. We estimated the association between participation in gardening and mental health outcomes (depression and anxiety subscale scores specified as continuous variables) using multivariable linear regression models.

To assess the robustness of our findings to potential confounding by unobserved variables, we conducted an e-value analysis (VanderWeele and Ding, 2017). The e-value identifies the minimum strength of association, on the risk ratio scale, that an unobserved confounder would need to have with both the exposure and the outcome in order to completely explain away the estimated association. A larger e-value suggests that an unobserved variable would need to pose very strong confounding in order to invalidate the findings.

## 3 Results

### 3.1 Characteristics of the sample

The sample included 242 family caregivers. Most [182 (75%)] were women. Most participants [181 (75%)] were children of the patients, and they reported having provided care for a mean duration of 8.4 years (S.D., 2.6). They reported significant caregiving burden with a mean ZBI of 56.9 (S.D, 12.9). Their patients had severe functional impairment, as suggested by the mean BADLS of 43.6 (S.D, 10.2). Approximately one-half of the sample reported severe or extremely severe symptoms of depression (105 [43%]) and anxiety (146 [60%]).

Slightly less than one-half (111 [46%]) did not participate in gardening, while 131 (54%) participated in gardening. Men comprised a larger proportion of those who participated in gardening activities compared with those who did not participate in gardening (39% vs. 8%; χ = 30.7, P < 0.001) (Table 1). Those who participated in gardening activities also reported lower levels of the symptoms of depression (12.0 vs. 31.6; t = 24.2, P < 0.001) and anxiety (11.9 vs. 29.9, t = 23.0, P < 0.001).

**Table 1:** Characteristics of the sample (N = 242).

	Did not participate in gardening			Participated in gardening			
	Freq.	Mean/Prop.	SD	Freq.	Mean/Prop.	SD	P-value
DASS depression subscale		31.6	7.93		12.03	4.41	<0.001
DASS anxiety subscale		29.9	7.41		11.9	4.68	<0.001
DASS stress subscale		33.1	7.16		13.7	5.05	<0.001
Caregiver age, years		45.4	15.5		43.6	14.3	0.35
Caregiver sex (female)		0.92	0.27		0.61	0.49	<0.001
Caregiver education, years		4.80	4.42		5.38	4.65	0.32
Caregiving duration, years		8.92	2.30		8.04	2.79	0.009
ZBI, total score		62.1	9.88		52.6	13.6	<0.001
BADLS, total score		47.7	6.87		40.2	11.2	<0.001
Patient age, years		87.4	8.72		85.7	8.92	0.14
Patient sex (female)		0.85	0.36		0.82	0.38	0.64
Patient’s relationship with caregiver							
Grandparent	18	0.16		18	0.14		0.32
Parent	85	0.77		96	0.73		
Spouse/other	8	0.07		17	0.13		

Notes: BADLS, Bristol Activities of Daily Living Scale; DASS, Depression, Anxiety, and Stress Scales; SD, standard deviation; ZBI, Zarit Burden Interview. P-values refer to those associated with t-tests (for continuous variables) and chi-squared tests (for categorical and dichotomous variables)

### 3.2 Association between participation in gardening activity and depression, anxiety, and stress

In a regression model with a single explanatory variable, participation in gardening had a statistically significant negative association with depression symptom severity (b = -19.6; 95% CI, –21.2 to −17.9) and explained 71% of the variance in the outcome. After adjustment for covariates, the estimated association between participating in gardening and depressive symptoms remained statistically significant (b = -18.4; 95% CI, −20.5 to −16.3) (Table 2).

**Table 2:** Estimated associations between participation in gardening activity and depression, anxiety, and stress.

	Depression	Anxiety	Stress
	Unstandardized coeff (95% confidence interval)		
Gardening	−18.4 (-20.5 to −16.3) ***	−16.6 (-18.6 to −14.6) ***	−18.6 (-20.6 to −16.6) ***
Caregiver age, years	0.06 (-0.02 to 0.13)	0.07 (-0.003 to 0.14)	0.06 (-0.01 to 0.12)
Caregiver sex (female)	1.40 (-0.61 to 3.41)	1.47 (-0.49 to 3.43)	1.71 (-0.26 to 3.68)
Caregiver education, years	0.06 (-0.16 to 0.28)	0.03 (-0.18 to 0.24)	0.04 (-0.16 to 0.25)
ZBI, total score	0.11 (0.05 to 0.17) ***	0.11 (0.05 – 0.17) ***	0.06 (0.0004 – 0.12) *
BADLS, total score	−0.04 (-0.13 to 0.05)	−0.01 (-0.10 to 0.07)	−0.03 (-0.12 to 0.06)
Patient age, years	−0.04 (-0.15 to 0.06)	−0.07 (-0.17 to 0.03)	−0.11 (-0.21 to 1.43) *
Patient sex (female)	0.03 (-2.08 to 2.14)	0.42 (-1.51 to 2.35)	−0.66 (-2.75 to 1.43)
Patient's relationship with caregiver			
Grandparent	Ref	Ref	Ref
Parent	−2.24 (-4.84 to 0.37)	−1.84 (-4.55 to 0.88)	−1.97 (-4.54 to 0.61)
Spouse/other	−0.06 (-3.88 to 3.76)	−1.29 (-5.05 to 2.48)	−1.81 (-5.47 to 1.86)
Constant	27.8 (18.09 – 37.54) ***	26.2 (16.7 – 35.6) ***	38.1 (28.9 – 47.3) ***

Notes: BADLS, Bristol Activities of Daily Living Scale; ZBI, Zarit Burden Interview; * p < 0.05; ** p < 0.01; *** p < 0.001.

Similarly, when we fitted a regression model with anxiety as the dependent variable and gardening as the single explanatory variable, participation in gardening had a statistically significant negative association with anxiety symptom severity (b = –18.0; 95% CI, −19.7 to −16.4) and explained 69% of the variance in the outcome. After adjustment for covariates, the estimated association between participating in gardening and anxiety symptoms remained statistically significant (b = -16.6; 95% CI, −18.6 to –14.6).

Finally, when fitting a regression model specifying stress as the dependent variable and gardening as the single explanatory variable, we found that participation in gardening had a statistically significant negative association with symptom of stress severity (b = -19.4; 95% CI, −21.0 to −17.8) and explained 72% of the variance in the outcome. After adjustment for covariates, the estimated association between participating in gardening and anxiety symptoms remained statistically significant (b = -18.6; 95% CI, −20.6 to –16.6). In all three regression models, the only other explanatory variables that had statistically significant associations with the outcomes were caregiving burden (measured by the ZBI) and the age of patient.

### 3.3 Sensitivity analysis

We explored the robustness of our findings to potential confounding by unobserved variables. In the multivariable regression analysis of depression symptoms, after converting the estimated regression coefficient to a standardized effect size, we calculated an e-value for the point estimate of 5.65 and an e-value for the confidence interval of 4.25; in the analysis of anxiety symptoms, we calculated an e-value for the point estimate of 5.49 and an e-value for the confidence interval of 4.13. Finally, in the analysis of stress, we calculated an e-value for the point estimate of 5.76 and an e-value for the confidence interval of 4.33. These e-values suggest that only very strong confounding by an unobserved variable could shift the estimated regression coefficients to include b = 0 or to even shift the associated confidence intervals to include 0.

## 4 Discussion

In this cross-sectional study of family caregivers of people living with dementia in a rural region of southwestern Uganda, we found that participation in gardening activity was associated with low symptoms of depression, anxiety, and stress. The estimated association was robust to covariate adjustment for caregiving burden and functional impairment. A sensitivity analysis indicated that the estimated association was also robust to confounding by unobserved variables, such that any unmeasured covariates would need to have a very strong association with both gardening activity and the mental health outcomes in order to shift the confidence interval around the estimated regression coefficient to include zero.

The finding that caregivers who reported participating in gardening activities generally reported low levels of mental health problems is consistent with existing literature suggesting that participation in gardening activities may contribute to improved mental wellbeing (Evans et al., 2016, Gonzalez and T., Patil, G., Martinsen, E., & Kirkevold, M. , 2011, Kam and Siu, 2010, Klemmer et al., 2005, Soga et al., 2017). Recent developments in agriculture and health suggest that daily contact with nature and green space has beneficial long-lasting effects on mental health (Barton and Rogerson, 2017, Kelley et al., 2017, Takano et al., 2002), including stress and anxiety symptoms (Beyer et al., 2014). One possible explanation for our findings is that involvement in gardening instills hope and optimism through the act of caring for crops and providing a source of future orientation in the anticipation of the harvest season (Cutillo et al., 2015, Hale et al., 2011). Secondly, involvement in gardening presents various elements of physical activity that have been shown to reduce an individual’s perception of stress and to improve overall mental health (Stults-Kolehmainen and Sinha, 2014). Another body of literature has shown improvement in health states among individuals who are involved in gardening compared with those that are not. For example, a recently published systematic review showed that those who participated in gardening had better health, as well as increases in general health and life satisfaction, compared with those who did not (Soga et al., 2017).

### 4.1 Limitations

We note that the cultural context of the present study significantly differs from the few existing studies on the mental health benefits of gardening which are based on data collected in high income countries, where participation in gardening is largely regarded as a voluntary leisure activity, unlike in large areas of sub-Saharan Africa, where participating in gardening activity is often essential for livelihood (Park et al., 2009, Rodiek, 2002, Van Den Berg and Custers, 2010). In the context of the present study, where food insecurity (for example) is widely prevalent, it is possible that food insecurity could confound our estimates: food insecurity has been linked to depression and mental health problems in diverse settings across sub-Saharan Africa (Dewing et al., 2013, Tsai et al., 2012, Tsai et al., 2016b, Weaver and Hadley, 2009) and people who are food insecure may be more likely to engage in gardening activity. However, our estimated e-values indicate that the association between food insecurity and symptoms of depression, anxiety or stress (and between food insecurity and participation in gardening activity) would need to exceed 4–5 on the risk ratio scale in order to completely explain away the estimated associations. In a recently published *meta*-analysis, the pooled association between food insecurity and depression was consistent across studies and statistically significant, but did not exceed an adjusted odds ratio of 2; moreover, the pooled estimate for the association between food insecurity and anxiety was not statistically significant (Pourmotabbed et al., 2020). Thus we are reasonably confident that our inability to adjust for food insecurity, or other indicators of socioeconomic status like household asset wealth (Smith et al., 2020, Smith et al., 2019), is unlikely to have biased our estimates to such a degree that the estimated associations would be completely explained away by such a variable. Future research studies in Africa should focus on longitudinal and/or experimental designs in order to replicate our core finding.

### 4.2 Conclusion

In this cross-sectional study of family caregivers of people living with dementia in rural Uganda, caregivers’ involvement in gardening activity was associated with lower severity of depression, anxiety and stress symptoms. If replicated, our work suggests a potential intervention that is likely to be both culturally acceptable and potentially effective for improving mental well-being in this high-risk population.

## Ethics approval and consent to participate

Ethical approval for the study was given by the Mbarara University of Science and Technology. We also received clearance to conduct the study from the Uganda National Council for Science and Technology and the Research Secretariat in the President’s office.

## Availability of data and material

The data sets used and analyzed during the current study are available from the corresponding author on request.

## Funding

Herbert Ainamani was supported through the Innovative Methods and Metrics for Agriculture and Nutrition Action (IMMANA) programme, led by the London School of Hygiene & Tropical Medicine (LSHTM). IMMANA is co-funded with UK Aid from the UK government and by the Bill & Melinda Gates Foundation INV-002962 / OPP1211308. Under the grant conditions of the Foundation, a Creative Commons Attribution 4.0 Generic License has already been assigned to the Author Accepted Manuscript version that might arise from this submission.’

## Authors’ contributions

HEA participated in the conception and design of the study, collected the data, performed data analyses, interpreted the data, and drafted the manuscript. WMB participated in the conception of the study, supervised data collection, and revised the manuscript. GZR, ST, RMK and EMB participated in the conception of the study and revised the manuscript. ACT participated in the conception of the study, supervised data analysis, and provided substantial revision of the manuscript. All authors read and approved the final manuscript.

## Declaration of Competing Interest

The authors declare that they have no known competing financial interests or personal relationships that could have appeared to influence the work reported in this paper.
